# Continuity and Change in the Arbëreshë Wild Food Plant Foraging in Inland Southern Italy

**DOI:** 10.3390/plants15132073

**Published:** 2026-07-03

**Authors:** Andrea Pieroni, Mousaab Alrhmoun, Irfan Ullah, Avni Hajdari, Ani Bajrami, Raivo Kalle, Naji Sulaiman, Renata Sõukand

**Affiliations:** 1 University of Gastronomic Sciences, Piazza Vittorio Emanuele II 9, 12042 Pollenzo, Italy; 2 Department of Medical Analysis, Tishk International University, Erbil 4001, Iraq; 3 Faculty of Agricultural, Environmental and Food Sciences, Free University of Bolzano, Piazza Università 5, 39100 Bolzano, Italy; 4 Department of Molecular Wood Biotechnology and Technical Mycology, Georg-August University of Göttingen, Büsgenweg 2, 37077 Göttingen, Germany; irfan.ullah@uni-goettingen.de; 5 Department of Biology, Faculty of Mathematical and Natural Science, University of Prishtina, Mother Theresa St, 10000 Prishtinë, Kosovo; avni.hajdari@uni-pr.edu; 6 Museum of Natural Sciences “Sabiha Kasimati”, University of Tirana, Sheshi Nënë Tereza 4, 1010 Tiranë, Albania; 7 Estonian Literary Museum, 51003 Tartu, Estonia; 8 Biocultural Diversity Lab, Department of Environmental Sciences, Informatics and Statistics, Ca’ Foscari University of Venice, Via Torino 155, 30172 Venezia, Italy

**Keywords:** Arbëreshë, wild food plants, ethnobotany, traditional ecological knowledge, Calabria, linguistic heritage

## Abstract

This study investigates the ethnobiology of wild food plants in Arbëreshë (Albanian-speaking) and neighbouring Calabrian communities in north-eastern Calabria, inland southern Italy. It examines how traditional ecological knowledge, plant use patterns, and cultural perceptions are represented across two datasets, contributing to the understanding of biocultural dynamics in Mediterranean rural contexts. Fieldwork was conducted through forty-six semi-structured interviews in five villages in north-eastern Calabria, Southern Italy. Data were compared with an ethnobotanical dataset collected in the Vulture area (northern Lucania, southern Italy) during 2000–2001. The comparison is treated as cross-spatial and diachronic at the level of observed ethnobotanical records. Because the study areas differ in ecological and socio-economic conditions, comparisons are presented as descriptive contrasts rather than as direct temporal change. Taxa were classified by citation frequency, and comparisons were conducted at genus level to describe patterns of presence and variation in reported wild plant use. A total of 82 wild food taxa were documented. The dataset was dominated by vascular plants, with frequent representation of the families Asteraceae, Brassicaceae, Apiaceae, and Lamiaceae. Arbëreshë participants reported 60 genera, including seven genera not recorded in the comparative dataset (*Asphodeline*, *Pimpinella*, *Hirschfeldia*, *Silene*, *Bellevalia*, *Leontodon*, and *Crocus*). Calabrian participants reported 28 genera, including three not recorded among Arbëreshë participants (*Clinopodium*, *Suillus*, and *Urospermum*). Twenty-one genera were present in both datasets. Differences in citation frequency and genus composition are observed between datasets, with variation across groups and contexts. The results show a shared set of commonly reported wild food taxa across datasets, alongside variation in less frequently reported genera. The findings describe differences in ethnobotanical records across communities and time-separated datasets, reflecting combined influences of ecological context, sampling conditions, and local knowledge practices.

## 1. Introduction

The study of Mediterranean ethnobiology of wild food plants, particularly in relation to minority and secluded rural communities, provides a compelling lens for examining the intricate relationships between cultural practice and the natural world [1,2,3]. The Arbëreshë, descendants of Albanian migrants who settled in southern Italy mainly during the fifteenth and sixteenth centuries, maintain a rich cultural heritage that encompasses distinctive linguistic features, folklore, and traditional ecological knowledge [4,5]. Their ethnobiological heritage, which includes the use of wild food and medicinal plants, reflects a long-standing adaptation to local Mediterranean ecosystems, and the persistence of ancestral practices within secluded rural settings continues to offer critical insights into biocultural resilience and adaptation [6,7,8]. Recent comparative ethnobotanical research conducted among Arbëreshë and neighbouring Calabrian communities further highlights how cultural and ecological continuity coexist with local diversification in plant-use patterns, demonstrating the resilience and dynamism of Arbëreshë traditional knowledge [5,9,10].

Our previous study showed that the Arbëreshë have preserved a distinct body of traditional knowledge regarding the collection, preparation, and consumption of wild plants, reflecting a deep understanding of local ecological systems embedded in cognitive and linguistic structures, as well as sensory foodscapes and culinary identities [4]. This knowledge extends beyond mere subsistence, embodying cultural values and a strong sense of identity. Comparing Arbëreshë ethnobotanical practices with those of neighbouring (autochthonous) South Italian communities sheds light on different “views” and perceptions in the use of local biodiversity [11].

Within the broader fields of ethnobiology, such comparative approaches are essential for understanding how human groups classify, use, and perceive biodiversity, and how these practices respond to ecological and socio-cultural variables and their diachronic changes, including linguistic shifts [12,13,14]. Cross-cultural ethnobotanical studies reveal patterns of change and resilience, demonstrating that traditional ecological knowledge evolves dynamically in response to environmental, socio-cultural, economic, and political factors [15,16,17]. These studies also highlight the significant contribution of local knowledge systems to biodiversity conservation and sustainable resource management, offering valuable lessons for contemporary challenges such as habitat loss, climate change and food insecurity, and underscore the importance of diachronic observations by communities over extended periods [16,18,19].

Mediterranean ethnobotany, as well as the Arbëreshë case, presents a prominent opportunity to explore the intersection of cultural persistence and human ecological adaptation, since traditional knowledge of wild food plants represents both biocultural heritage and a practical resource for sustainability. Therefore, investigating how Arbëreshë communities forage wild species can inform community-based conservation and reinforce local identity in a rapidly changing rural landscape [20]. An earlier ethnobotanical study conducted in 2000–2001 among Arbëreshë communities in Northern Lucania documented the central role of wild greens (“*liakra*”) in local food systems and cultural identity. The present study builds on that historical dataset to explore how similar ethnobotanical knowledge persists or changes across space and time. In fact, despite our previous ethnobotanical work among the Arbëreshë, little has been done in the past two decades on this aspect of the South Italian ethnobiology, and we still do not know if and how Arbëreshë wild food plant knowledge and practices have persisted, adapted, or transformed in inland Southern Italy. A methodological caveat should be acknowledged: the 2000–2001 and 2025 datasets derive from two Arbëreshë regions separated by approximately 200 km and characterised by partially distinct ecological and socio-economic contexts. Therefore, the present comparison should not be interpreted as a strictly longitudinal assessment, but rather as a cross-spatial diachronic comparison that allows exploration of broad trajectories of continuity and change in local ecological knowledge. To assess this, it is crucial to better understand the shifts in healthy, wild, and local plant food systems and their associated human ecologies over the past few decades in rural, remote regions that are equally rich in biological and cultural diversity and that have historically been considered an important component of traditional Mediterranean diets. This study aims to document and compare traditional knowledge and use of wild food plants among Arbëreshë and Calabrian-speaking communities in northeastern Calabria. By integrating these 2025 field data with our earlier records from 2000–2001, the study aims to investigate the continuity and transformation of Arbëreshë foraging practices. It reflects on how socioecological changes and linguistic persistence or loss may have influenced temporal shifts in foraging and culinary practices for wild food plants. By combining ethnobotanical, human ecological, and linguistic perspectives, this study aims to deepen our understanding of the resilience and adaptation of this Mediterranean biocultural food heritage within small historical linguistic enclaves.

## 2. Methods

**Figure 1:**
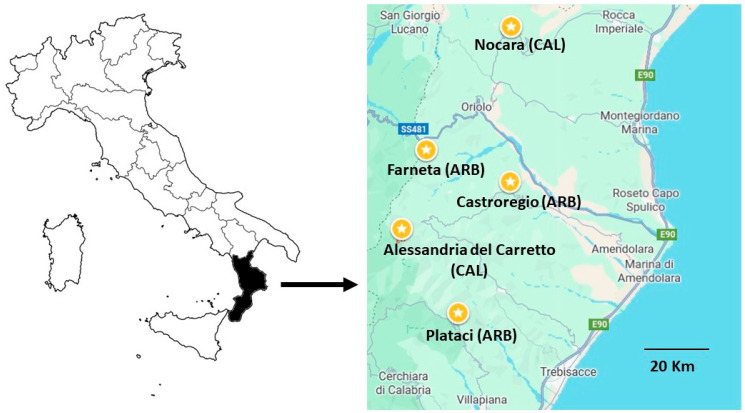
The study area and the visited villages. CAL: Calabrian-speaking villages; ARB: Arbëreshë-speaking villages.

Forty-six participants were interviewed in March 2025 in five villages in north-eastern Calabria, Southern Italy (Figure 1). The study sites included Arbëreshë-speaking communities (Plataci, Castroregio, Farneta) and Calabrian-speaking communities (Alessandria del Carretto, Nocara). Sites were selected based on (i) documented presence of Arbëreshë cultural traits in some communities and predominantly Calabrian identity in others, and (ii) geographical isolation relative to major coastal centres along the Ionian Calabrian coast. Ecological and socio-economic characteristics of the Vulture (2000–2001) and north-eastern Calabria (2025) study areas are summarised in Supplementary Table S1 to contextualise comparisons between datasets. For comparability, the historical 2000–2001 dataset was reconstructed from the original ethnobotanical field records collected in the Arbëreshë villages of Ginestra, Maschito, and Barile (Vulture area). The original survey included 68 interviews conducted using semi-structured interviews, free-listing, participant observation, and botanical specimen verification. All historical records were harmonised to genus level prior to comparative analysis.

**Figure 2:**
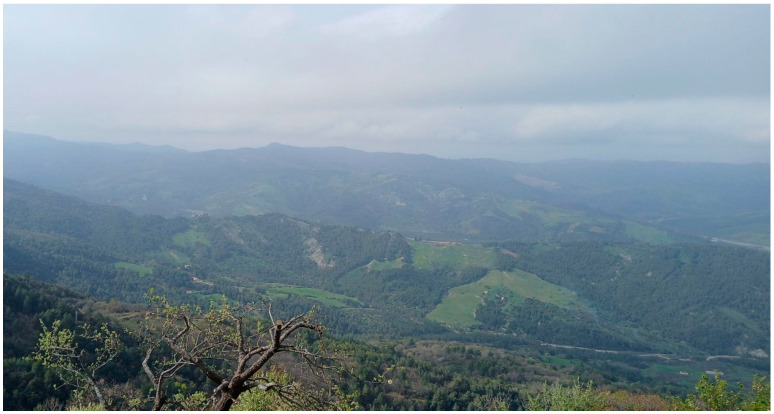
The landscape of the study site surrounding Nocara.

The inner Ionian Calabria is characterised by a rugged and stratified landscape that reflects centuries of ecological adaptation and human persistence. Steep slopes descend from the Aspromonte and Serre mountains toward narrow coastal plains, creating a mosaic of micro-environments shaped by geology, altitude, and exposure. Terraced olive groves and vineyards (increasingly abandoned), along with occasional small patches of vegetable gardens, punctuate a predominantly forested terrain of Mediterranean maquis, chestnut, and oak forests (Figure 2).

**Figure 3:**
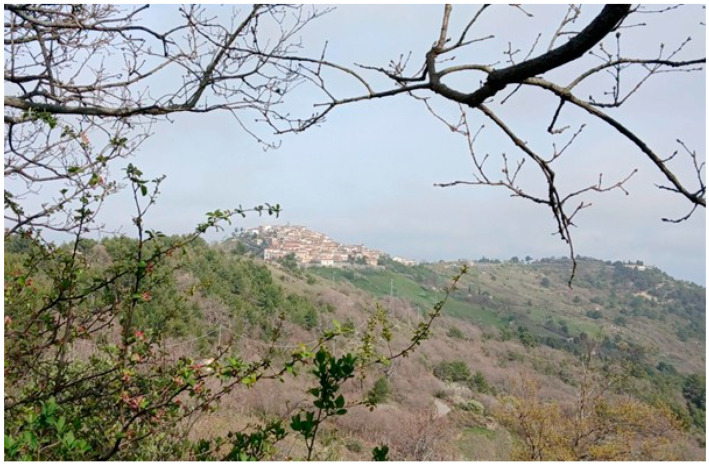
The village of Castroregio.

Scattered hamlets and villages (resembling ghost settlements since they are mainly uninhabited) are often perched on ridges (Figure 3) or bear witness to an ancient settlement pattern oriented toward self-sufficiency. The ecological gradient between the uplands and the Ionian coast fosters exceptional biodiversity, including endemic plant species and a few still existing signs of traditional agro-ecosystems. Yet this landscape is also marked by processes of depopulation, land abandonment, and erosion. The research focused on elderly locals and individuals connected to agropastoral life. The sample consisted of 46 participants aged between 50 and 90 years, with a near-balanced sex distribution. Participants were selected using purposive and snowball sampling. Selection targeted individuals identified as knowledgeable about wild food plant gathering, particularly those with experience in agropastoral activities or household food preparation. The study was designed to document ethnobotanical knowledge rather than to produce statistically representative population estimates. Interviews were conducted in Italian or Albanian. Semi-structured interviews focused on wild and semi-domesticated plants used as food, including wild greens, seasonings, fruits, snacks, and infusions. Foraging was defined as the collection of wild or semi-wild plant and fungal resources from unmanaged or minimally managed environments. Cultivated taxa were included only when their local use differed from common agricultural or market uses. Free-listing protocols were used and aligned with those applied in the 2000–2001 dataset to enable comparison between datasets.

Very few cultivated taxa, whose local food utilisation diverged from the mainstream food uses, were considered as well. The field protocols based on free-listing were aligned with those of the 2000–2001 dataset to enable diachronic comparison. Each folk taxon was identified by collecting specimens where available, consulting *Flora* *d’Italia* and its online portal (https://dryades.units.it/floritaly/index.php, accessed on 1 June 2026), comparing folk names with previously collected specimens, or hypothetically attempted by analysing locals’ descriptions of plant ecology, morphology, and sensory characteristics; digital photographs or drawings were used to confirm identification when necessary. Not all taxa could be verified through physical specimen collection. In a minority of cases, identification relied on folk names, verbal descriptions, and visual confirmation through photographs. These records were retained only when identification confidence was sufficiently high, but they remain a methodological limitation. Botanical nomenclature follows World Flora Online, and mycological nomenclature follows Index Fungorum (https://www.indexfungorum.org/names/names.asp, accessed on 1 June 2026), with the plant family assignments adhering to the Angiosperm Phylogeny Website Version 14 (https://www.mobot.org/mobot/research/apweb/, accessed on 1 June 2026). Herbarium specimens are deposited at Ca’ Foscari University of Venice (UVV), bearing codes UVVETBOTCALARB01-34. The local folk plant names were reported following the standards of written Albanian and (Northern Calabrian) Italian, respectively; the neutral, mid-central vowel schwa, which is present in both languages, was rendered by the sign “ë”. Ethical approval was granted by the Ethics Committee of the University of Gastronomic Sciences (code 29042024), in accordance with the International Society of Ethnobiology Code of Ethics. Oral informed consent was obtained before the interviews and recordings.

### Data Analysis

To ensure comparability between datasets, analyses were conducted at the genus level. This approach was used to reduce potential variation related to species-level ecological availability. Apparent differences in genus presence between datasets are treated as differences in recorded ethnobotanical observations rather than as the disappearance or emergence of taxa. Analyses focused on wild green genera, as wild fruits and teas were not consistently recorded in the 2025 dataset. Diversity was assessed using Shannon–Wiener (H’) and Simpson (D) indices for two Arbëreshë datasets. Shannon’s index describes the richness and evenness of recorded taxa, while Simpson’s index describes the dominance structure in citation patterns. Local plant names were organised into a bilingual lexicon, and the proportion of Albanian- and Italian-derived names was quantified descriptively. All analyses were conducted in R (v. 4.3.3) and SAS 9.4. The vegan package was used for diversity indices, iNEXT for rarefaction, Indicspecies for indicator analysis, FactoMineR for multivariate ordination, and ggplot2 for visualisation. No inferential statistical tests were performed. Citation frequencies, diversity indices, and multivariate ordinations were used exclusively as descriptive and exploratory tools to summarise ethnobotanical patterns within and between datasets. Accordingly, all comparisons are interpreted descriptively rather than as tests of statistical significance.

## 3. Results

### 3.1. Overview of Foraged Wild Food Plants in NE Calabria

In 2025, a total of 82 taxa were recorded in the study area, including 68 plant taxa and a limited number of additional fungal taxa, along with two plant and one mushroom taxa unidentified (Table 1), representing 62 genera distributed across more than 30 botanical families. Among these, vascular plants dominated, with the most represented families being Asteraceae, Brassicaceae, Apiaceae, and Lamiaceae.

**Figure 4:**
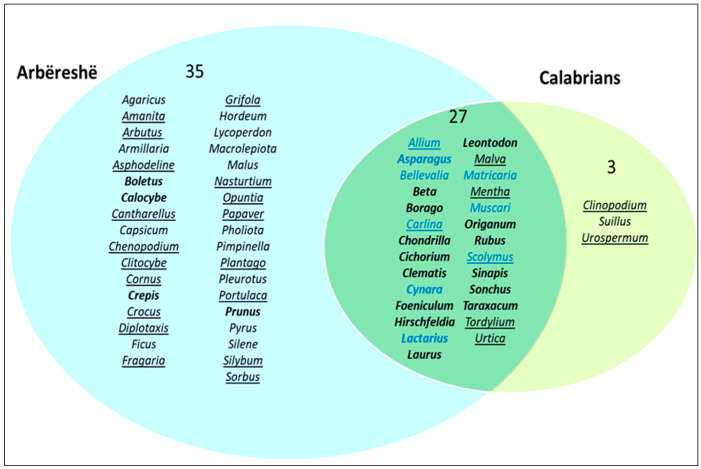
Overlaps of the wild food genera quoted by the two studied communities in NE Calabria in 2025. Genera marked in bold have been very commonly cited (VC), those in regular fonts have been widely quoted (C), and those underlined have been rarely quoted (R); genera in blue have shared (or similarly sounding) folk names.

In contrast, macrofungi were mainly found in the families Agaricaceae, Boletaceae, and Pleurotaceae. The most foraged botanical families are characterised by strong tastes (bitter, pungent, and aromatic), suggesting that these ingredients in local Mediterranean rural cuisines are crucial drivers of specific sensory and aesthetic qualities that are appreciated. Considering the community-specific patterns, the Arbëreshë reported 60 genera of wild plants and mushrooms, 35 of which were unique to their community and 25 shared with neighbouring Calabrian groups, which had only three unique genera (Figure 4). The marked asymmetry in genus diversity suggests that the Arbëreshë have retained a broader repertoire of wild-food knowledge, likely reflecting both stronger cultural transmission and the persistence of traditional foraging practices within a minority context, while still sharing a common biocultural foundation with neighbouring populations.

**Table 1:** Recorded foraged wild food taxa and their local food uses.

Scientific Name (Herbarium Number)	Family	Recorded Folk Name(s) Among Arbëreshë	Recorded Folk Name(s) Among Calabrians	Used Parts	Detailed Local Food Use	Frequency of Quotation
*Agaricus campestris* L.	Agaricaceae	Venlia		Fruiting body	Cooked	C
*Allium ampeloprasum* L. (CALARB25)	Amaryllidaceae	Purriqë *	Puorrë	Whole plants ^WG^	Seasoning	R
*Amanita caesarea* (Scop.) Pers.	Amanitaceae	Ovulë *		Fruiting body	Raw, cooked	R
*Arbutus unedo* L.	Ericaceae	Marëze, Marlëzë		Fruits	Snack	R
*Armillaria mellea* (Vahl) P. Kumm.	Physalacriaceae	Dashlia *		Fruiting body	Cooked	C
*Asparagus acutifolius* L. (CALARB08)	Asparagaceae	Speraognë *, Spërëgnë *, Sporognë *, Spuranghë *	Sario, Sparachë	Young shoots ^WG^	Omelettes, boiled, also with noodles	VC
*Asphodeline lutea* Rchb.	Asphodelaceae	Pichkumarë		Shoots ^WG^	Boiled	R
*Beta vulgaris* L. (CALARB03)	Amaranthaceae	Seskillë, Seskulë	Veta	Aerial parts ^WG^	Sauteed, sauteed and pan-fried, filled pasta	VC
*Boletus* spp.	Boletaceae	Porchinë *		Fruiting body	Cooked	C
*Borago officinalis* L.	Boraginaceae	Fendados	Borraina	Leaves ^WG^	Sauteed, sauteed and pan-fried	C/R
*Calocybe gambosa* (Fr.) Singer	Lyophyllaceae	Ordinatë * malit		Fruiting body	Cooked	VC
*Cantharellus cibarius* Fr.	Hydnaceae	Muklia		Fruiting body	Cooked	R
*Capsicum annuum* L.	Solanaceae	Pebër		Fruits	Cooked, dried (pebër krushkë) and cooked, dressing for noodles (with olive oil: chif)	VC
*Carlina* spp.	Asteraceae	Karlinë *	Karlinë	Flower receptacles ^WG^	Snacks	R
*Chenopodium album* L. (CALARB21)	Amaranthaceae	Llabbottë		Leaves ^WG^	Sauteed, sauteed and pan-fried	R
*Chenopodium bonus-henricus* L.	Amaranthaceae	Spinakë		Leaves ^WG^	Sauteed, sauteed and pan-fried	R
*Chondrilla juncea* L. (CALARB15)	Asteraceae	Bresazë, Bretheza	Incudine	Shoots ^WG^	Salads	VC
*Cichorium intybus* L., *Leontodon hispidus* L.	Asteraceae	Shkoirë	Cicoria	Whorls ^WG^	Sauteed, sauteed and pan-fried, soup with wild fennel and pork rind	VC
*Clematis vitalba* L. (CALARB19)	Ranunculaceae	Kuipri, Kurprë, Kurpri	Grabullinë	Young shoots ^WG^	Omelettes, boiled, soups (with garlic and chilli: pishës)	VC
*Clinopodium nepeta* (L.) Kuntze	Lamiaceae		Chersullë	Aerial parts ^WG^	Tea: cardiotonic	R
*Clitocybe geotropa* (Bull.) Quél.	Clitocybaceae	Settembrine *		Fruiting body	Cooked	R
*Cornus mas* L.	Cornaceae	Thanë		Fruits	Snack	R
*Crepis bursifolia* L., (CALARB02) *Crepis mollis* (Jacq.) Asch., (CALARB27)	Asteraceae	Chikorië, Shkoirë, Shkoria		Whorls ^WG^	Sauteed, sauteed and pan-fried, soup with wild fennel and pork rind	VC
*Crocus* spp.	Iridaceae	Kaxha		Bulbs ^WG^	Snack	R
*Cynara cardunculus* L. (CALARB34)	Asteraceae	Kaichoffë *, Karchofë *	Carciofë	Flower receptacles, peeled stems ^WG^	Cooked; pickles	VC
*Diplotaxis erucoides* (L.) DC.	Brassicaceae	Lapsanë		Whorls ^WG^	Sauteed, sauteed and pan-fried	R
*Ficus carica* L. (CALARB05)	Moraceae	Fiku		Pseudofruits (fiqë)	Raw, dried, decoction (ribollitë)	C
*Foeniculum vulgare* Mill. (CALARB11)	Apiaceae	Mraj embël	Fenucchië	Aerial parts ^WG^	Sauteed, sauteed and pan-fried, soups (menesthrë)	VC
				Fruits	Seasoning for sausages	VC
*Fragaria vesca* L.	Rosaceae	Dretha		Fruits	Raw	R
*Grifola frondosa* (Dicks.) Gray	Grifolaceae	Dashlja		Fruiting body	Cooked, pickled	R
*Hirschfeldia incana* (L.) Lagr. -Foss.	Brassicaceae	Rapë	Lapsanë	Young shoots ^WG^	Sauteed, sauteed and pan-fried	VC
*Hordeum vulgare* L.	Poaceae	Elbi		Fruits	Decoction (ribollitë)	C
*Lactarius deliciosus* (L.) Gray	Russulaceae	Kumbarinë	Kumarinë	Fruiting body	Cooked	VC
*Laurus nobilis* L. (CALARB10)	Lauraceae	Dhafënë	Laurë	Leaves	Seasoning	VC
*Leontodon hispidus* L. (CALARB26)	Asteraceae	Shkoirë	Cikoria	Whorls ^WG^	Sauteed, sauteed and pan-fried, soup with wild fennel and pork rind	VC
*Lycoperdon* sp.	Lycoperdaceae	Fendauikë		Fruiting body	Cooked	R
*Macrolepiota procera* (Scop.) Singer	Agaricaceae	Vallia		Fruiting body	Cooked	C
*Malus domestica* (Suckow) Borkh.	Rosaceae	Mollë		Fruits	Decoction (ribollitë)	C
*Malva sylvestris* L. (CALARB01)	Malvaceae	Mëllëgë	Mavalë	Aerial parts ^WG^	Tea: bellyaches in children	R
*Matricaria recutita* L. (CALARB24)	Asteraceae	Ankamillë *, Hamomille *	Agumillë	Flowering tops	Decoction (ribollitë): hypertension, digestive (kids)	C
*Mentha* spp. (incl *Mentha aquatica* L. CALARB23)	Lamiaceae	Menderse, Mëndrësë		Leaves ^WG^	Seasoning, tea	R
*Bellevalia romana* (L.) Sweet CALARB31) *Muscari comosum* (L.) Mill. CALARB29), *Muscari commutatum* Guss.	Asparagaceae	Chipollinë *	Cipolline	Bulbs ^WG^	Cooked, pickled	C
*Nasturtium officinale* R.Br.	Brassicaceae	Shelpurë		Whorls ^WG^	Sauteed, sauteed and pan-fried	R
*Opuntia ficus-indica* (L.) Mill.	Cactaceae	Fiqë turkë		Fruits	Raw	R
*Origanum vulgare* L.	Lamiaceae	Rigannë	Rigan	Flowering tops ^WG^	Seasoning	VC
*Papaver rhoeas* L.	Papaveraceae	Lulëkuqë		Whorls ^WG^	Sauteed, sauteed and pan-fried	R
*Pholiota aegerita* (V. Brig.) Quél.	Tubariaceae	Pioklia		Fruiting body	Cooked	C
*Pimpinella anisoides* V. Brig.	Apiaceae	Mraj embël		Seeds	Seasoning for taralli	C
*Plantago lanceolata* L.	Plantaginaceae	Liukalios		Leaves	Tea	R
*Pleurotus ostreatus* (Jacq.) P. Kumm.	Pleurotaceae	Pinellia *		Fruiting body	Cooked	C
*Portulaca oleracea* L.	Portulacaceae	Burdulakë		Aerial parts ^WG^	Salads	R
*Prunus cerasifera* Ehrh.	Rosaceae	Kumëlla e egër		Fruits	Raw, jams	VC
*Prunus cerasus* L.	Rosaceae	Zhershia, Zirshi		Fruits	Raw, jams	VC
*Prunus spinosa* L.	Rosaceae	Kollumbri		Fruits	Snack	R
*Pyrus communis* L. (CALARB06)	Rosaceae	Dhardhë		Fruits	Decoction (ribollitë)	C
*Pyrus pyraster* (L.) Burgsd.	Rosaceae	Gorricë		Fruits	Snack	R
*Rubus fruticosus* L. (CALARB13)	Rosaceae	Fërezit	Rua	Fruits (mënzë/civezë)	Raw, jams	VC
*Scolymus hispanicus* L.	Asteraceae	Kardunxhellë *	Cardongello	Mydribs ^WG^	Boiled	R
*Silene vulgaris* (Moench) Garcke (CALARB32)	Caryophyllaceae	Bathulka, Bathurza		Young shoots ^WG^	Boiled	C
*Silybum marianum* (L.) Gaertn.	Asteraceae	Gliombi		Mydribs ^WG^	Boiled	R
*Sinapis alba* L. (CALARB09)	Brassicaceae	Rapë, Cimë *	Lapsanë	Young shoots ^WG^	Sauteed, sauteed and pan-fried	VC
*Sonchus oleraceus* L. (CALARB14)	Asteraceae	Rrishellë	Cardellë	Whorls ^WG^	Sauteed, sauteed and pan-fried	VC
*Sorbus domestica* L.	Rosaceae	Vadhëza		Fruits	Raw	R
*Suillus luteus* (L.)	Suillaceae		Bavoso	Fruiting body	Sauteed, sauteed and pan-fried	C
*Taraxacum officinalis* F.H.Wigg. (CALARB12)	Asteraceae	Bochka	Cicoria	Whorls ^WG^	Sauteed, sauteed and pan-fried, soup with wild fennel and pork rind	VC
*Tordylium apulum* L. (CALARB20)	Apiaceae	Gasmuljerë, Grasmullire	Rampulline	Whorls ^WG^	Sauteed, sauteed and pan-fried	R
*Urospermum dalechampii* (L.) Scop. (CALARB16)	Asteraceae		Cikoria coglinutë	Whorls ^WG^	Sauteed, sauteed and pan-fried	R
*Urtica dioica* L. (CALARB04)	Urticaceae	Hithi	Ardigola	Leaves ^WG^	Sauteed, sauteed and pan-fried	R
Unidentified fungal taxon		Peshakane *		Fruiting body	Cooked	C
Unidentified plant taxon		Liuta farducha		Leaves ^WG^	Sauteed, sauteed and pan-fried	R
Unidentified plant taxon		Diegsi		Leaves ^WG^	Sauteed, sauteed and pan-fried	R

VC = very commonly quoted taxa; C = commonly quoted taxa; R = rarely quoted taxa. ^WG^ = taxa locally classified as wild greens (“*liakra*”/“foglie”); * folk names quoted by Albanians but having an Italian origin.

Among Arbëreshë participants, most reported taxa retained Albanian-derived folk names, whereas Calabrian participants used exclusively Italian/Calabrian nomenclature. This linguistic asymmetry supports the interpretation of bilingual plant naming as a cultural buffer preserving ethnobotanical memory. Wild plants were used in diverse culinary applications, including boiled or sautéed aerial parts, and, more rarely, in omelettes, soups, pasta fillings, and the consumption of fruits (raw, in jams, or as decoctions). Seasonings and teas were also reported for specific taxa, such as *Foeniculum*, *Origanum*, *Pimpinella*, and *Plantago* spp. The Arbëreshë community showed a remarkably greater diversity of wild-food plant knowledge and a still-resilient use of Albanian folk plant names (Figure 4). In contrast, the Calabrians used exclusively South Italian/Calabrian folk plant names (Table 1). The reasons for this remarkable difference in the ethnobotanical knowledge between Albanians and Calabrians could be various. While it is striking that Albanian-origin communities tend to forage a much wider range of wild food taxa than their Calabrian neighbours, despite sharing the same ecological setting, this difference cannot be simply explained by resource availability, as both groups inhabit similar landscapes and have comparable access to the same foraging environments. Instead, it may reflect a more profound cultural attachment to traditional cuisine and local identity. Among the Albanians of NE Calabria, especially in Plataci, the collection and preparation of wild greens remain integral to everyday domestic life and are perceived as markers of continuity with ancestral foodways. In this respect, the socio-economic peripherality and marginality of Plataci village helped preserve local knowledge. Additionally, Plataci had a significant recent history of migration in Switzerland and Germany over the past decades. This wave went partially back home in recent years: Return migration may contribute to the persistence of foraging practices through nostalgia, culinary memory, and renewed engagement with traditional food heritage. In contrast, among the autochthonous Calabrians, foraging has become a more sporadic and symbolic activity, often practised for culinary nostalgia or leisure rather than sustenance. This contrast highlights how culinary memory and cultural pride can sustain ecological practices even amid social and economic change. The persistence of foraging among the Albanians of NE Calabria thus represents not merely an adaptive strategy, but a form of cultural resilience, maintaining a living connection between landscape, identity, and taste. Fungal foraging should be considered separately from wild plant gathering because it follows distinct ecological, seasonal, and cultural dynamics. The presence of Italian-derived names for some mushrooms among Arbëreshë participants does not necessarily indicate post-migration acquisition of fungal knowledge, since mushroom foraging has historically existed across Albanian cultural regions as well. Instead, bilingual nomenclature may reflect lexical borrowing, shifting cultural salience, or recent revalorization of mushroom foraging.

### 3.2. Diachronic Changes in Arbëreshë Wild Food Plants

**Figure 5:**
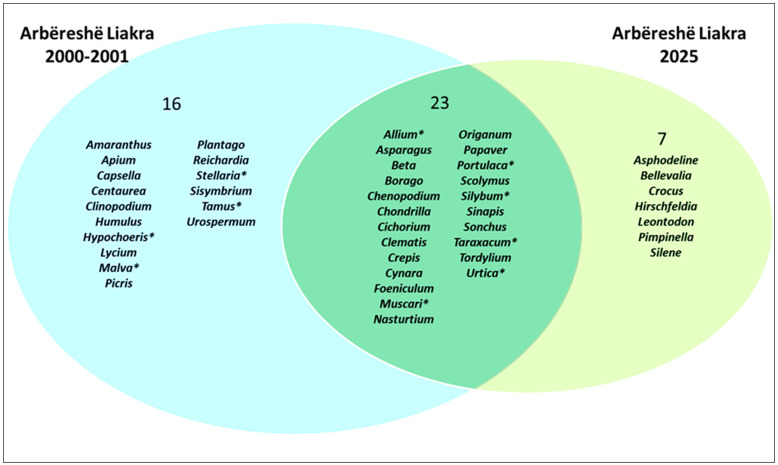
Venn diagram showing continuity, loss, and novel wild green genera foraged by Arbëreshë communities based on 46 interviews in 2025 and the historical 2000–2001 dataset. * refers to recorded genera as memory and is no longer used in 2000–2001.

The comparison between the 2000–2001 North Lucania dataset and the 2025 Calabria survey highlights both continuity and change in Arbëreshë wild-greens (*liakra*) practices (Figure 5). Of the genera recorded in 2000–2001, 16 genera are no longer cited in 2025, including *Amaranthus*, *Apium*, *Capsella*, *Centaurea*, *Clinopodium*, *Humulus*, *Hypochoeris*, *Lycium*, *Malva*, *Picris*, *Plantago*, *Reichardia*, *Stellaria*, *Sisymbrium*, *Tamus*, and *Urospermum*. These “disappeared” or, possibly, very sporadic plant reports refer mainly to synanthropic taxa, generally found and foraged along countryside roads and field margins, suggesting that these environments may have become less frequented or of minor importance for contemporary foraging. Their disappearance may also indicate a shift away from collecting wild greens as a side activity of small-scale farming, which was previously done in vineyards, olive orchards, around vegetable gardens, or while shepherding. Additionally, their absence may reflect a generational discontinuity, as fewer and fewer elderly and mid-generation community members in our 2025 field study appear to be actively managing gardens or olive orchards.

In contrast, seven genera (*Asphodeline*, *Pimpinella*, *Leontodon*, *Hirschfeldia*, *Silene*, *Bellevalia*, and *Crocus*) appear exclusively in the 2025 survey, possibly reflecting specific peculiarities of the study site customs or recently adopted foraging trends. A few of these taxa may have been likely incorporated into the local food heritage repertoire for their particular, attractive, very distinctive pungent, bitter, or aromatic taste (*Hirschfeldia*, *Leontodon*, and *Pimpinella* spp. respectively), or may function as a culinary substitute (*Asphodeline* sp.) for wild asparagus according to several interview narratives, whose gathering is increasing constantly and may risk to become soon unsustainable in many areas of Southern Europe, or possibly for newly gained popularity of specific plants via modern written foraging or cooking guides and manuals (*Silene* sp.).

A core of 23 genera persisted across both time and regional differences, suggesting some cultural and ecological continuity. Especially interesting is the presence of many plants that were “just remembered” in the 2000–2001 dataset, but are actively used in the 2025 dataset. Specific taxa (especially *Asparagus*, *Chondrilla*, *Cichorium*, *Foeniculum*, *Origanum*, *Scolymus*, *Sinapis*, and *Sonchus* spp.) have played, and continue to play, a central culinary role in Arbëreshe heritage. In particular, the 18 genera reported as used in 2000–2001 and still today represent a cultural core of the Arbëreshë subsistence biocultural local food system and identity.

The comparison of shared wild-food genera between 2000–2001 and 2025 (Figure 5) reveals both continuity and subtle shifts in Arbëreshë foraging practices. Among the 23 genera consistently cited in both periods, 10 are now intensively used. Several genera demonstrated increased recognition or continuity in contemporary use. For instance, *Urtica dioica*, *Taraxacum officinale*, and *Portulaca oleracea*, which were only remembered in the 2001 dataset, are now reported as rarely foraged (R) in 2025. Likewise, *Muscari* spp. were only remembered in Lucania; they are currently collected and consumed in the study site, indicating that some historically recalled practices might have re-entered active use.

**Figure 6:**
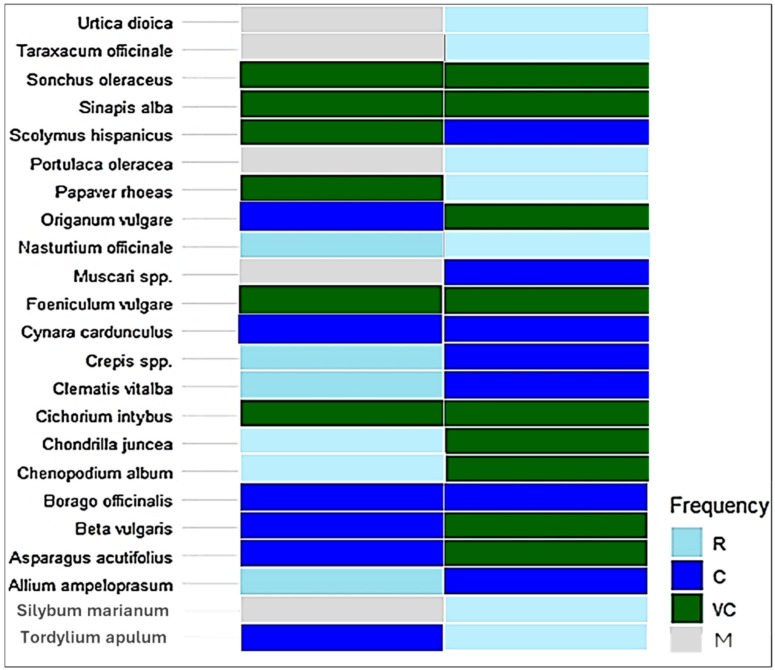
Comparison of citation frequency of Arbëreshë wild taxa 2000–2001 vs. 2025. Citation frequency categories: R = Rare, C = Common, VC = Very Common, M = In memory.

Other genera exhibit stable patterns or only minor changes. *Foeniculum vulgare*, *Sinapis alba*, *Sonchus oleraceus*, and *Cichorium intybus* remained among the most frequently cited taxa (VC) across both periods, confirming their persistent role in the Arbëreshë foraging repertoire. In contrast, *Cynara cardunculus* maintained a common citation pattern (C), consistent with its sporadic yet enduring culinary use. A few taxa showed declines or more variable reporting. *Papaver rhoeas* and *Nasturtium officinale* are now cited as rare (R) rather than very common, while *Borago officinalis* shifted from being commonly cited to more variably reported (C/R). These patterns may indicate reduced reliance on specific taxa or diversification in wild green preferences. The inclusion of *Silybum* (recorded only as memory in 2001 but occasionally foraged in 2025) and *Tordylium* (common in 2001 and rare in 2025) illustrates how plant knowledge can persist even as usage frequency changes (Figure 6).

**Figure 7:**
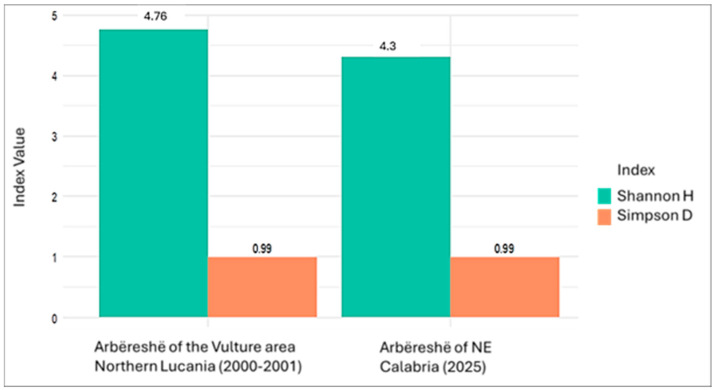
Shannon and Simpson Indices of foraged wild plants among the Arbëreshë of the Vulture area (Northern Lucania, 2000–2001) vs. the Arbëreshë of NE Calabria (2025).

Overall, these findings reveal nuanced transformations in foraging practices over the past two decades. While several core genera continue to anchor Arbëreshë ethnobotanical traditions, others have gained or lost prominence, influenced by shifting culinary tastes, ease of collection, and contemporary food culture. This evolving pattern suggests a gradual transition from primarily subsistence-oriented foraging toward more gastronomic and identity-driven engagement with wild foods. The comparison of wild plant diversity between the Vulture area in 2001 and the Arbëreshë communities in Calabria in 2025 confirms the observed expansion in foraged taxa and highlights significant diachronic changes in ethnobotanical knowledge. The Arbëreshë Calabria dataset shows higher species richness in agreement with higher Shannon and Simpson diversity indices (Figure 7). These indices indicate not only a broader taxonomic spectrum but also a more even distribution of plant use, suggesting that no single species dominates the local repertoire. This quantitative pattern supports the qualitative findings of increased diversity among edible taxa and reflects a more balanced and diversified engagement with wild food resources. Overall, the results point to a dynamic evolution of Arbëreshë foraging practices, shaped by both cultural continuity and the adoption of new taxa, leading to a richer, more evenly structured ethnobotanical system than that documented in Vulture two decades earlier.

## 4. Discussion

The diachronic comparison presented here has important limitations. The 2000–2001 dataset originated from the Vulture area of Northern Lucania, whereas the 2025 dataset derives from north-eastern Calabria. Although both areas are inland southern Italian Arbëreshë territories, they differ in elevation, habitat composition, land-use history, and socio-economic trajectories. Therefore, differences observed between datasets may reflect temporal change, spatial heterogeneity, or both. Reported expansion in taxa richness should consequently be interpreted cautiously, as it may partly derive from ecological availability or sampling differences rather than genuine expansion of knowledge. Our comparative analysis of Arbëreshë and neighbouring Calabrian wild-food knowledge (2000–2001 vs. 2025) reveals three interlinked patterns regarding wild greens. First, a resilient core of commonly used wild greens has persisted across two decades, reflecting continuity in traditional foraging practices. Second, selective losses were observed, particularly among ruderal and hedgerow greens, as well as taxa associated with forest margins. Third, the 2025 Calabrian dataset showed notable expansion along with modest shifts in the parts used and culinary applications. Quantitatively, species richness and diversity were higher in the 2025 dataset compared to the 2000–2001 Vulture area.

### 4.1. Arbëreshë Resilience and Changes

The persistence of a core set of wild greens such as *Cichorium intybus*, *Borago officinalis*, *Urtica dioica*, and *Asparagus acutifolius* mirrors earlier ethnobotanical accounts among Arbëreshë and other Southern Italian populations, where a stable “*liakra*/wild greens” repertoire remains central to seasonal diets and household cuisines [4,21]. This continuity likely reflects several reinforcing mechanisms: deep intergenerational transmission of collection and preparation know-how [22,23]; strong culinary embedding, since these greens are incorporated into everyday dishes rather than restricted to ritual or medicinal contexts; and ecological fit, as many of these taxa thrive in abundant, edge, or anthropogenic habitats that have persisted despite broader landscape changes [24].

Comparable patterns of durable leafy vegetable repertoires have been emphasised in previous Arbëreshë and southern Mediterranean studies, where wild “*liakra*” serve as key markers of identity and culinary resilience [4,21,25]. The persistence of this core thus illustrates how cultural memory and ecological stability jointly sustain traditional plant use even amid demographic, linguistic, and environmental transformation.

The disappearance or decline of some ruderal and hedgerow taxa may reflect changes in land use and reduced interaction with marginal agroecosystems. Regional land-use reports suggest a progressive decline in agricultural activity and expansion of shrub and forest cover in both Basilicata and Calabria over the past two decades. Such ecological transitions may reduce the abundance and visibility of ruderal edible taxa while increasing woodland habitats. These broader ecological trends are summarized in Supplementary Table S1. One reason for this may be the rapid abandonment of small-scale farming over the past few decades. This pattern echoes broader trends documented in depopulated Mediterranean and European landscapes, where agricultural abandonment often triggers shrub encroachment or forest expansion, resulting in the loss of ruderal niches [26], as well as gentrification and over-tourism, as we recently described in the case of Corfu [3].

These landscape-level transformations diminish encounter rates and the day-to-day visibility of formerly common edibles, leading to reduced citation frequencies among younger informants and, ultimately, knowledge attrition [27]. Social factors further compound these ecological processes: younger generations’ evolving food habits, greater reliance on marketed food items, and limited time for foraging make species that once served routine subsistence functions less salient [28]. This dual dynamic, land abandonment and sometimes ecological unavailability combined with profound socio-cultural changes, is widely recognised across studies, as summarized by a recent review on local environmental knowledge linked to foraging [20], and is corroborated by our own observations that elders in our field study area often recalled specific taxa no longer known or actively used by younger cohorts.

The emergence or increased reporting of fungi in 2025 suggests a revival of forest-based foraging practices and the possible rise in new culturally salient ingredients, due to the pleasure of collecting fungal taxa for the family for only a few days each year, mainly in the fall, or even for minor economic incentives [29]. Two interlinked mechanisms appear to underlie this trend: increasing demand and the gastronomic valorisation of wild resources. Across Europe, foraging has been revalorised by chefs, local festivals, and gastronomic tourism, stimulating renewed interest in mushrooms and wild aromatics and generating both market outlets and prestige value for these products [30,31]. Contemporary studies consistently highlight the growing popularity and commercialisation of edible fungi and wild ingredients in the 21st century, often linked to rural tourism, niche food markets, and broader sustainability discourses. Such valorisation processes can prompt renewed harvesting efforts and the conscious re-learning of species identification and use [32].

Parallel ecological shifts have likely reinforced these cultural dynamics. The abandonment of marginal fields, followed by afforestation or shrub encroachment, has expanded forest cover and structural complexity in many areas, increasing the local abundance of woodland fungi and shrubs. These changes simultaneously enhance the availability of forest taxa while diminishing the abundance of ruderal and hedgerow species. This dual, habitat-mediated turnover closely aligns with our observed pattern of losses and gains [27]. Taken together, these processes suggest that the 2025 ethnobotanical repertoire reflects both ecological opportunity (through the expansion of forest-type habitats) and cultural revaluation (through gastronomy and tourism), culminating in an extended and partially transformed foraging landscape. Alternative explanations for this habitat turnover should also be considered, including shifts in culinary preferences, differential sampling, and local ecological heterogeneity between study regions.

The persistence of Arbëreshë plant names, together with the rise in Arbëreshë–Italian bilingual recordings, indicates ongoing linguistic mediation of plant knowledge and the emergence of hybrid transmission pathways. This pattern aligns with earlier findings that Arbëreshë lexical elements remain embedded in local plant nomenclature and medicinal terminology even in contexts of intense contact with Italian dialects [33]. Bilingual naming frequently reflects processes of cultural diffusion, identity maintenance, and the adaptive reshaping of folk nomenclature in response to evolving social settings [34].

Such bilingual transmission can function as a cultural buffer: it safeguards older linguistic forms while facilitating the uptake of plant knowledge by younger Italian-speaking generations. This process is often reinforced through intergenerational and community activities such as local festivals, school projects, markets, and mixed-language family networks where plant uses and terminologies are reframed and revitalised [11]. The coexistence of Arbëreshë and Italian terms thus illustrates how linguistic hybridity supports the continuity of traditional ecological knowledge amid cultural change.

### 4.2. Implications for Local Food Biocultural Heritage, Its Conservation and Valorisation Policies

The persistence of a resilient core of wild edibles across two decades, combined with the expansion of taxa and uses in NE Calabria in 2025, highlights both continuity and adaptive innovation within Arbëreshë and neighbouring Calabrian food knowledge systems. The quantitative analyses suggest that linguistic and village-based differentiation is limited, while overall diversity and evenness were higher in 2025. This pattern may indicate cumulative knowledge exchange and shared regional adaptation rather than fragmentation. These results underscore the value of community-based conservation programs that strengthen intergenerational transmission through workshops, school gardens, and community validation events [16,35,36,37,38,39,40].

Documenting recipes, seasonal calendars, and bilingual plant names can safeguard intangible biocultural heritage while promoting sustainable, context-specific use [6,41,42,43].

Because observed losses and gains may be partly associated with habitat differences and land-use change, with ruderal taxa declining and forest-associated taxa expanding, landscape management emerges as a key conservation priority. Maintaining small-scale farming systems and the mosaic of hedgerows, riparian strips, pastures, and managed woodlands supports both the ecological and cultural spectrum of wild edibles, sustaining biodiversity and biocultural diversity [44,45,46]. The rise in fungi and aromatic taxa in 2025 reflects not only ecological opportunity but also changing cultural economies. These trends can be harnessed as locally led, sustainable livelihood options through guided foraging, value-added products, and community festivals if harvesting remains ecologically sound and benefits are equitably shared [37,42,43,47,48].

Finally, the continued transmission of plant knowledge in both languages indicates that linguistic diversity remains integral to ethnobotanical resilience, as the persistence of local languages often underpins the maintenance, transmission, and contextual meaning of traditional ecological knowledge [9,49,50,51,52,53].

Supporting the Arbëreshë language through bilingual educational materials and participatory documentation of plant names and uses can reinforce identity while enhancing accessibility for non-Arbëreshë participants. Such integrated approaches linking habitat management, cultural revitalisation, and language preservation offer a pathway toward sustaining both ecological and cultural resilience in these evolving Mediterranean landscapes.

### 4.3. Limitations of the Study

While this study provides valuable insights into the persistence and transformation of wild plant knowledge, it also has some limitations. The two considered areas for the comparative analysis, although having a similar vegetation, climate and cultural realm, are distant about 200 km and are not identical, especially in terms of the socio-economic development they had during the past two-three decades: while the Vulture area (home of the field study of 2000–2001) has seen the development of the automotive industry and the gentrification of wine production, NE Calabria (home of the field study presented here) remained pretty economically depressed and peripheral.

Integrating ethnobotanical surveys with ecological monitoring, including an in-depth assessment of land-use change, would strengthen causal inference about habitat-driven shifts in plant and mushroom availability and, consequently, plant/fungal uses. Additionally, targeted in-depth investigations of the socioeconomic drivers, such as household income, food procurement pathways, economic structure of the communities, the effect of tourism, and the persistence of household other (non-foraged-based) traditional domestic food practices, and how these may be linked to culinary identities, could clarify the influence of social factors on the nature and transmission of local food knowledge. A further limitation concerns sampling. Since interviews targeted knowledgeable participants rather than a random population sample, the dataset reflects retained expert knowledge rather than average community knowledge. Therefore, present-day erosion of traditional ecological knowledge at the broader population level may be greater than documented here.

## 5. Conclusions

This study revisited, after more than two decades, the ethnobotanical and ethnomycological heritage of the Arbëreshë minority of inland Southern Italy, providing a diachronic and cross-cultural comparison with both historical data (2000–2001, Vulture area) and neighbouring Calabrian communities (2025). The results reveal a remarkable persistence of a cultural core of wild food plants, particularly pungent, bitter and aromatic taxa, which continue to structure local diets and seasonal practices. This continuity confirms that foraging continues to serve as a vital expression of identity, memory, and ecological adaptation within the Arbëreshë world, and that the traditional *liakra* repertoire remains a living, evolving component of the Mediterranean Diet.

At the same time, several transformations emerged. The disappearance of ruderal and hedgerow taxa, once associated with subsistence and small-scale agropastoral life, reflects ecological and socio-economic shifts, including land abandonment, forest expansion, and demographic ageing of foragers. These processes have reduced the visibility and perceived relevance of several formerly common taxa while simultaneously supporting the re-emergence of woodland-associated resources. The findings suggest a possible transition from primarily subsistence-oriented gathering toward identity-, heritage-, or pleasure-oriented engagement with wild foods. These intertwined ecological and cultural processes illustrate how local environmental knowledge flexibly responds to changing landscapes and social meanings.

Quantitative diversity analyses confirmed these trends, showing greater taxonomic richness and evenness in 2025 compared with 2000–2001. Multivariate exploratory analyses did not reveal strong clustering by language group or village, suggesting substantial overlap in foraging patterns across communities. This suggests that nature knowledge exchange between Arbëreshë and Calabrian neighbours may be ongoing, leading to a regionally integrated, hybrid ethnobotanical system. Importantly, bilingual folk nomenclature persists among the Arbëreshë (especially in Plataci), revealing both linguistic resilience and creative adaptation. The coexistence of Albanian and Italian names encapsulates how language contact can reinforce rather than erode traditional knowledge, serving as a bridge between generations and cultural groups.

Overall, this research demonstrates that the Arbëreshë ethnobiology of wild foods exemplifies both resilience and renewal. The continuity of core taxa and practices embodies deep temporal stability. At the same time, the selective adoption of new species and uses reveals an adaptive response to ecological availability, economic incentives, and changing cultural tastes. In this sense, the Arbëreshë landscape emerges as a biocultural laboratory where past and present coexist, and where traditional food knowledge remains dynamic rather than fossilised.

Future research should extend this diachronic framework by integrating ecological field monitoring, assessments of land-use changes, and socio-economic analyses of emerging wild-food resource economies. Such approaches can illuminate how biodiversity, cultural memory, and sustainability interact in peripheral Mediterranean contexts. Ultimately, sustaining both biological and cultural diversity in these marginal areas requires reinforcing intergenerational transmission of nature-related knowledge, sustaining small-scale farming, promoting linguistic diversity, and maintaining the fine-grained mosaic of interconnected human ecologies that has long supported the *liakra* world.

## Data Availability

The original contributions presented in this study are included in the article/Supplementary Material. Further inquiries can be directed to the corresponding author.

## Supplementary Materials

The following supporting information can be downloaded at https://www.mdpi.com/article/10.3390/plants15132073/s1: Supplementary Table S1: Ecological, socio-economic, and methodological context of the 2000–2001 and 2025 datasets; Table S2. Comparison of the sampling characteristics and field protocols of the historical (2000–2001) and contemporary (2025) ethnobotanical surveys.
