# Supplementary material for: Continuity and Change in the Arbëreshë Wild Food Plant Foraging in Inland Southern Italy
Source: Plants (Basel). 2026 Jul 3;15(13):2073. doi: 10.3390/plants15132073 (PMC13364172; doi:10.3390/plants15132073)
Supplement: Supplementary file 1 [file plants-15-02073-s001.zip › plants-4402273-supplementary.pdf]

Supplementary Methods and Supporting Information

This supplementary document provides additional methodological details regarding dataset harmonisation, sampling characteristics, ecological context, bilingual lexical classification, and descriptive analytical procedures.

Supplementary Table S1

Ecological, socio-economic, and methodological context of the 2000–2001 and 2025 datasets

Dimension	2000–2001 dataset (Vulture, Northern Lucania)	2025 dataset (NE Calabria)	Data type / source	Use in this study
Geographic setting	Inland Southern Italy (Vulture area)	Inland Southern Italy (north-eastern Calabria)	Field site description	Contextual comparison of two regions
Elevation range	Not systematically recorded in original dataset	50–900 m (participant villages range)	Field observation / site metadata	Environmental context only
Sampling design	Semi-structured interviews + free listing	Semi-structured interviews + free listing	Ethnobotanical field methods	Ensures methodological comparability
Participant selection	Informal selection via local knowledge holders (farming/pastoral background)	Purposive + snowball sampling of knowledge holders	Ethnographic recruitment	Defines knowledge-focused sampling
Sample size	Not explicitly standardised in original publication (reconstructed dataset used)	46 participants	Field records	Descriptive context only
Age range	Not systematically reported	50–90 years	Interview metadata	Describes informant profile
Sex distribution	Not systematically reported	Approximately balanced	Interview metadata	Descriptive context only
Primary livelihoods	Small-scale agriculture, pastoralism, household subsistence	Mixed livelihoods; reduced agropastoral activity reported	Regional ethnographic literature + field observation	Socio-economic background description

Land-use structure	Mosaic of cultivated fields, orchards, and marginal land	Increased abandonment of terraces and marginal agriculture	Regional land-use literature	Contextual environmental description only
Vegetation structure	Mixed agroecosystems with hedgerows and field margins	Mosaic of forest, shrub expansion, and abandoned terraces	Regional ecological reports	Environmental background context
Dominant land-use trend	Active small-scale farming (historical baseline)	Reduced cultivation and increased land abandonment	Secondary literature	Contextual comparison only
Data collection tools	Semi-structured interviews, free listing	Semi-structured interviews, free listing	Ethnobotanical protocol	Ensures methodological alignment
Taxonomic resolution used in analysis	Species-level originally collected; harmonised to genus level for comparison	Recorded and analysed at genus level	Data harmonisation step	Ensures comparability
Cultivated taxa inclusion	Included if locally used outside mainstream agricultural context	Same criterion applied	Ethnobotanical classification rule	Standardised inclusion rule
Linguistic context	Arbëreshë + Italian dialect contact zone	Arbëreshë + Calabrian Italian contact zone	Field linguistic observation	Context for naming analysis
Bilingual naming coding	Not systematically quantified in original dataset	Albanian vs Italian-derived names coded per genus record	Coding procedure applied to 2025 dataset	Used only for descriptive proportions
Land-use data source	Regional ethnographic literature (post-hoc contextualisation)	Regional land-use reports and ecological descriptions	Secondary sources only	Contextual framing only
Statistical use of context variables	Not analysed statistically	Not analysed statistically	Not applicable	No modelling performed
Role in study	Comparative ethnobotanical baseline	Contemporary ethnobotanical dataset	Primary data	Used for descriptive comparison only

Supplementary Table S2. Comparison of the sampling characteristics and field protocols of the historical (2000–2001) and contemporary (2025) ethnobotanical surveys

Characteristic	2000–2001 Vulture (Northern Lucania)	2025 NE Calabria
Study period	April–June 2000; August and November 2000; March–July 2001	March 2025
Study area	Arbëreshë villages of Ginestra, Maschito, and Barile	Arbëreshë villages of Plataci, Castroregio, and Farneta; Calabrian-speaking villages of Alessandria del Carretto and Nocara
Cultural groups surveyed	Arbëreshë	Arbëreshë and neighbouring Calabrian communities
Sample size	68 participants	46 participants
Participant profile	Local knowledge holders retaining traditional environmental knowledge	Local knowledge holders retaining traditional ecological knowledge
Age profile	62 of 68 participants (>91%) were older than 50 years	50–90 years
Main occupation/background	Primarily families maintaining strong links with farming and traditional agricultural activities	Elderly residents and individuals connected with agropastoral life
Sampling strategy	Purposive selection of knowledgeable informants	Purposive and snowball sampling of recognised knowledge holders
Interview methods	Semi-structured interviews, structured questionnaires (Ginestra), individual and group interviews	Semi-structured interviews
Ethnobotanical protocol	Participant observation, free-listing, botanical specimen verification, accompanied plant collection	Free-listing, botanical specimen verification where possible, photographs and verbal descriptions when necessary
Botanical identification	Fresh specimens, field herbarium, guided plant collection	Fresh specimens, Flora d'Italia, herbarium comparison, photographs and field observations
Main food categories recorded	Wild food plants, with emphasis on wild greens and food preparations	Wild greens, seasonings, fruits, snacks, selected cultivated taxa with non-mainstream food uses
Taxonomic level used in the present comparison	Harmonised to genus level	Genus level

Purpose in the present study	Historical reference dataset	Contemporary dataset

A. Historical dataset (2000–2001): fieldwork and comparability

The historical dataset was derived from ethnobotanical fieldwork conducted between April 2000 and July 2001 in the Arbëreshë villages of Ginestra, Maschito, and Barile (Vulture area, Northern Lucania, Southern Italy). The original study included 68 interviews with local knowledge holders, most of whom (62 participants) were older than 50 years and belonged to families maintaining strong connections with traditional agricultural activities.

Fieldwork combined participant observation, semi-structured and structured interviews, free-listing, individual and group interviews, and botanical verification using fresh specimens, field herbarium material, and accompanied plant collection whenever possible. Informants were selected through local contacts based on their recognised knowledge of traditional wild food plants, farming, pastoralism, and household food preparation.

For the present study, the historical dataset was harmonised with the 2025 dataset by updating botanical nomenclature according to World Flora Online and Index Fungorum and aggregating all records to the genus level. This harmonisation reduced potential bias arising from differences in local species composition between the two study areas. Because the original study predated current reporting standards, some metadata (e.g., detailed demographic characteristics) were not systematically recorded. Consequently, only variables available in both datasets were included in the descriptive comparison.

B. Sampling strategy (2025 dataset)

The 2025 survey included 46 participants recruited through purposive and snowball sampling. Informants were selected because they were recognised within their communities as knowledgeable regarding wild food gathering and traditional plant use.

Participants primarily represented individuals with direct experience in:

- household food preparation,
- agropastoral activities,
- traditional foraging practices.

The objective was to document retained traditional ecological knowledge rather than to obtain a statistically representative sample of the local population. Accordingly, no random or stratified sampling procedures were applied.

C. Data harmonisation and analytical framework

To maximise comparability between datasets, all records were harmonised prior to analysis by:

- updating botanical nomenclature using World Flora Online and Index Fungorum;
- aggregating all taxa to the genus level;
- including cultivated taxa only when their local food use differed from mainstream agricultural consumption;
- analysing plant and fungal taxa separately where appropriate.

Citation frequencies, diversity indices (Shannon–Wiener H′ and Simpson D), and exploratory multivariate ordinations were used exclusively to summarise patterns within and between datasets. These analyses were descriptive and exploratory and were not intended to test statistical hypotheses or establish inferential significance.
